# Prioritizing psychological relief: fast-absorbing collagen sutures improve parental well-being in pediatric facial trauma care

**DOI:** 10.3389/fped.2025.1628763

**Published:** 2025-08-11

**Authors:** Peili Xie, Kaixi Tan, Jianfei Zhang

**Affiliations:** ^1^ Department of Pediatrics, The Second Affiliated Hospital, Hengyang Medical School, University of South China, Hengyang, China; ^2^ Department of Burns and Plastic Surgery, The Affiliated Nanhua Hospital, Hengyang Medical School, University of South China, Hengyang, China; ^3^ Department of Burns and Plastic Surgery, The Second Affiliated Hospital, Hengyang Medical School, University of South China, Hengyang, China

**Keywords:** pediatric facial trauma, fast-absorbing collagen sutures, parental anxiety, family quality of life, scar cosmesis, family-centered care, postoperative psychological burden

## Abstract

**Objective:**

To assess the psychological status and quality of life improvements in parents of children undergoing facial trauma repair emphasizing cosmetic outcomes with fast-absorbing collagen sutures, and to compare these outcomes with those in parents of children repaired with nylon sutures. A secondary objective was to compare scar outcomes between the two suture materials.

**Methods:**

From January 2024 to October 2024, 120 primary caregivers (defined as the parent providing ≥80% of daily wound care) of pediatric facial trauma patients were randomized into an observation group (*n* = 60, 7-0 fast-absorbing collagen sutures, non-removal) or a control group (*n* = 60, 7-0 nylon sutures, removed on day 7). Psychological assessments included the Self-Rating Anxiety Scale (SAS), Self-Rating Depression Scale (SDS), and Generic Quality of Life Inventory-74 (GQOLI-74). Scar formation was evaluated using the Scar Cosmesis Assessment and Rating (SCAR) scale at 1 month postoperatively.

**Results:**

Baseline SAS/SDS scores and GQOLI-74 domain scores showed no intergroup differences (*P* > 0.05). Post-intervention, both groups exhibited reduced anxiety (ΔSAS: −12.3 ± 2.1 vs. −8.7 ± 1.8) and depression (ΔSDS: −10.9 ± 1.9 vs. −6.4 ± 1.5), with superior reductions in the observation group (*P* < 0.05). GQOLI-74 scores improved significantly in the observation group across all domains (psychological: 82.4 ± 5.3 vs. 75.1 ± 4.8; social: 79.6 ± 4.7 vs. 71.2 ± 4.1; *P* < 0.05). SCAR scores showed no significant differences between groups (3.07 ± 1.78 vs. 3.23 ± 1.64, *P* > 0.05).

**Conclusion:**

Fast-absorbing collagen sutures significantly alleviate parental anxiety/depression and improve family quality of life without compromising scar outcomes compared to nylon sutures. The non-removal protocol simplifies postoperative care, highlighting the importance of integrating caregiver psychological well-being into suture material selection.

## 1 Introduction

Pediatric facial trauma represents a prevalent emergency condition, accounting for 41.8% of childhood injuries according to the China National Injury Surveillance System (1). Contemporary management extends beyond functional restoration to emphasize aesthetic outcomes. The widespread adoption of suture techniques aimed at optimizing cosmetic results—particularly subcutaneous tension-reducing sutures combined with epidermal apposition—has become clinical consensus for minimizing scar formation risks (2).

However, postoperative care complexities impose multidimensional stress on parents. As primary caregivers, their anxiety and depressive states not only compromise personal well-being but may also negatively influence children's treatment adherence through emotional contagion, potentially triggering post-traumatic stress disorders (3). Notably, conventional non-absorbable sutures (e.g., nylon) requiring removal pose additional psychological burdens. The suture removal process, often accompanied by pediatric distress and wound re-exposure (4), may exacerbate parental anxiety, resulting in a “secondary trauma” effect.

Existing research predominantly focuses on the impact of suture materials on wound healing speed, infection rates, and scar assessment scales, while paying insufficient attention to parental psychological states (4). Although absorbable sutures are hypothesized to reduce homecare burden due to their non-removal, their mechanisms of action on caregiver mental health remain unclear: current literature lacks both comparative studies between different suture materials and exploration of dynamic associations between parental psychological stress and caregiving behaviors. This knowledge gap often leads to clinical decision-making processes that overlook family psychological support needs, hindering the achievement of family-centered trauma management objectives.

While sutures remain a cornerstone for complex pediatric facial lacerations, alternative wound closure methods have been explored to enhance child comfort. Tissue adhesives (e.g., Dermabond® (5) offer advantages such as rapid application and avoidance of needle-related distress, with studies reporting comparable cosmetic outcomes to sutures in selected cases. Adhesive strips (e.g., Steri-Strips™ (6) further provide a non-invasive option for low-tension wounds. However, existing comparisons primarily focus on technical outcomes (infection rates, scar cosmesis) or child-specific factors (pain during application), with limited attention to caregiver psychological burden. Crucially, no studies have quantified how suture material selection (e.g., absorbable vs. non-absorbable) affects parental mental health—despite the unique stress imposed by postoperative suture removal protocols in children. This gap is particularly relevant for wounds requiring layered closure, where sutures remain indispensable. Our study thus addresses a critical unmet need: evaluating whether suture material choice itself—independent of closure technique—can mitigate caregiver distress by eliminating removal procedures, while maintaining aesthetic outcomes. While our focus remains on comparing suture materials, we acknowledge that wound closure technique selection (e.g., sutures vs. adhesives) depends on multifaceted clinical factors including wound type and patient age. This study specifically addresses the understudied psychological impact of suture material properties within suture-indicated scenarios.

To our knowledge, this is the first study to directly quantify the impact of suture material selection (absorbable vs. non-absorbable) on parental mental health outcomes, bridging the gap between biomedical and psychosocial research in pediatric trauma care. Through a randomized controlled trial, therefore, this randomized controlled trial aims: (1) To assess and compare levels of parental anxiety, depression, and family quality of life following pediatric facial trauma repair using fast-absorbing collagen sutures vs. nylon sutures; (2) To compare scar outcomes assessed by the SCAR scale between the two suture material groups. We hypothesized that the simplified postoperative care associated with non-removable collagen sutures would be associated with reduced parental psychological distress and improved quality of life, without compromising scar cosmesis compared to removable nylon sutures. The findings may offer practical insights for suture material selection in pediatric facial trauma repair when sutures are indicated, and could inform the development of family-oriented psychological intervention strategies during the perioperative period.

## 2 Materials and methods

### 2.1 Study population

This prospective study enrolled pediatric patients undergoing facial trauma repair with emphasis on cosmetic outcome (using layered closure and precise epidermal approximation) repair for facial trauma at the Department of Plastic Surgery of The Second Affiliated Hospital, Hengyang Medical School, University of South China between January 2024 and October 2024. The study protocol was approved by the Institutional Review Board of The Second Affiliated Hospital, Hengyang Medical School, University of South China.

Inclusion Criteria: (1) Children aged ≤10 years with facial trauma involving skin, adipose tissue, fascia, or muscle layers (any gender/geographic origin). (2) Injury-to-treatment interval <12 h. (3) Wound characteristics: linear lacerations or minor contused lacerations with ≤1 cm tissue defects amenable to primary closure after debridement. (4) Parental capacity to complete written assessments.

Exclusion Criteria: (1) Parental history of psychiatric disorders. (2) Parental physical disabilities or major systemic diseases. (3) Parental cognitive impairments hindering communication/questionnaire completion.

Ancestral and Geographic Background:

Consistent with regional demographics, all enrolled families reported ≥3 generations of residence in mainland China with East Asian ancestry.

Demographic and psychological data were collected at the individual caregiver level. All analyses treated caregivers as independent observational units given:

(a) Single caregiver enrollment per child;
(b) No paired data from secondary caregivers;
(c) Research focus on direct care provider psychology.

The randomization was conducted using a simple randomization method with a computer-generated random number sequence (via SPSS 27.0). After enrollment, each caregiver was assigned a unique identifier, and the sequence was used to allocate participants to the observation (collagen sutures) or control (nylon sutures) group at a 1:1 ratio. To ensure allocation concealment, the randomization sequence was generated and managed by an independent researcher not involved in participant recruitment, surgical procedures, or outcome assessment.

### 2.2 Surgical methods

After thorough irrigation with copious normal saline, the wound and surrounding 10 cm area were disinfected with povidone-iodine solution. Sterile draping was applied, followed by local infiltration anesthesia using an appropriate amount of 2% lidocaine hydrochloride injection (Suicheng Pharmaceutical Co., Ltd.). For deeper wounds, hydrogen peroxide and normal saline were used to rinse away debris such as soil. Severely contused tissues (skin, adipose, and fascia) were excised using tissue scissors. The connective tissues on both sides of the wound were fully mobilized and released (approximately 0.5–1 cm in width), and the wound edges were trimmed to achieve clean margins, followed by another normal saline rinse. Hemostasis was achieved using electrocautery or suture ligation. Anatomical layers were carefully identified, and 5-0 absorbable surgical sutures (Johnson & Johnson Medical Shanghai Co., Ltd.) were used for layered closure from deep to superficial layers (muscle, adipose, fascia, and subcutaneous tissue). Inverting sutures were applied to the superficial adipose and upper third of the dermis to achieve tension-free closure of the outermost skin.

The observation group received 7-0 fast-absorbing collagen sutures (Shandong Boda Medical Products Co., Ltd.), which undergo complete absorption within 7–10 days post-implantation, this property eliminates the need for manual removal, while the control group received 7-0 nylon sutures (Johnson & Johnson Medical Shanghai Co., Ltd.) for precise epidermal alignment. This layered closure technique is employed to minimize tissue tension and optimize cosmetic outcome.

The skin was rinsed again with povidone-iodine solution and normal saline, coated with a small amount of erythromycin gel, and covered with sterile dressing under pressure bandaging.

### 2.3 Postoperative care

For wounds sutured with 7-0 nylon threads (control group), routine disinfection and dressing changes were performed every other day until suture removal on postoperative day 7. In contrast, wounds closed with 7-0 fast-absorbing collagen sutures (observation group) required no routine dressing changes. These patients underwent a single saline irrigation on postoperative day 7 to remove blood crusts, followed by standard wound monitoring.

### 2.4 Outcome measures

(1) Psychological Status: Parental anxiety and depression levels were assessed using the Self-Rating Anxiety Scale (SAS) (7) and Self-Rating Depression Scale (SDS) (8) preoperatively and 1 month postoperatively. Both scales contain 20 items each, with total scores ranging from 20 to 80 points. Higher scores indicate more severe anxiety or depressive symptoms.
(2) Quality of Life: The Generic Quality of Life Inventory-74 (GQOLI-74) (9) was administered preoperatively and 1 month postoperatively to evaluate four domains: physical health, psychological well-being, social functioning, and material living conditions. This 74-item questionnaire assigns domain-specific scores from 0 to 100 points, where higher values reflect better quality of life outcomes.
(3) Scar Formation: The scars were evaluated by Scar Cosmesis Assessment and Rating (SCAR) scale one month after surgical repair of facial emergency trauma. The total score was 15. The higher the score, the more obvious the scars were.

### 2.5 Statistical analysis

All statistical analyses were performed using SPSS 27.0 (IBM Corp.). Continuous variables are presented as mean ± standard deviation (x¯ ± s) and analyzed using Student's *t*-test for intergroup comparisons. Categorical data are expressed as frequency counts with percentages [*n* (%)] and assessed via chi-square test. A two-tailed *P*-value < 0.05 was considered statistically significant.

## 3 Results

### 3.1 Caregiver demographic characteristics

The results showed that there were no significant differences in characteristics such as age, gender, educational level, income level, and previous trauma care experience between the two groups of caregivers. This ensured the balanced comparability of the baseline characteristics of the samples, providing a solid foundation for the reliability of subsequent research results (Table 1).

**Table 1:** Caregiver demographic characteristics.

Characteristic	Nylon group (*n* = 60)	Collagen group (*n* = 60)	*P*-value
Age (years), mean ± SD	32.5 ± 4.8	33.1 ± 5.2	0.472
Gender, *n* (%)			0.318
Female (Mother)	54 (90.0%)	57 (95.0%)	
Male (Father)	6 (10.0%)	3 (5.0%)	
Education, *n* (%)			0.621
High school or lower	22 (36.7%)	19 (31.7%)	
College degree	31 (51.7%)	34 (56.7%)	
Postgraduate	7 (11.6%)	7 (11.6%)	
Monthly income (CNY), *n* (%)			0.538
<1,000	15 (25.0%)	12 (20.0%)	
1,000–1,500	32 (53.3%)	35 (58.3%)	
>1,500	13 (21.7%)	13 (21.7%)	
Previous trauma care experience, *n* (%)	18 (30.0%)	21 (35.0%)	0.557

Statistical tests: Independent *t*-test (age), Chi-square (categorical variables).

### 3.2 Comparison of psychological Status

#### 3.2.1 SAS scores

No significant intergroup difference was observed in baseline SAS scores (*P* > 0.05). Post-intervention, both groups exhibited reduced SAS scores compared to baseline, with the observation group demonstrating significantly lower scores than the control group (*P* < 0.05) (Table 2).

**Table 2:** Comparison of SAS scores between two groups before and after intervention [scores, ($\bar{x}$±s)].

Group	Before intervention	After intervention	*t*	*P*
Control	47.28 ± 4.42	35.41 ± 3.45	−2.624	0.022
Observation	47.19 ± 4.62	22.04 ± 2.67	−17.474	<0.001
t	0.163	−11.104		
P	0.878	<0.001		

#### 3.2.2 SDS scores

Baseline SDS scores showed no statistical difference between groups (*P* > 0.05). Following intervention, SDS scores decreased in both cohorts, with the observation group achieving markedly lower scores vs. the control group (*P* < 0.05) (Table 3).

**Table 3:** Comparison of SDS scores between two groups before and after intervention [scores, ($\bar{x}$±s)].

Group	Before intervention	After intervention	*t*	*P*
Control	43.92 ± 5.52	34.45 ± 4.81	8.745	<0.001
Observation	42.74 ± 5.11	23.36 ± 4.33	18.752	<0.001
t	0.557	11.105		
P	0.607	<0.001		

### 3.3 Quality of life assessment

#### 3.3.1 GQOLI-74 scores

Pretreatment GQOLI-74 domain scores were comparable between groups (*P* > 0.05). Postoperatively, the observation group showed statistically significant improvements across all four domains (physical, psychological, social, and material) compared to the control group (*P* < 0.05) (Table 4).

**Table 4:** Comparison of GQOLI-74 scores between two groups before and after intervention [scores, ($\bar{x}$±s)].

Time	Group	Physical function	Psychological function	Social function	Material life
Before intervention	Control	50.46 ± 8.25	41.70 ± 9.34	47.03 ± 8.53	36.63 ± 8.08
	Observation	51.39 ± 9.81	43.48 ± 8.86	46.76 ± 8.61	35.52 ± 7.78
	t	−0.126	−0.239	0.039	0.171
	P	0.906	0.823	0.971	0.872
After intervention	Control	60.51 ± 9.24	50.43 ± 9.23	54.77 ± 9.37	44.61 ± 8.52
	Observation	79.84 ± 8.30	64.28 ± 8.60	66.90 ± 8.40	55.88 ± 8.51
	t	−3.480	−7.115	−6.247	−6.065
	P	0.008	0.000	0.000	0.000

### 3.4 Comparison of scar formation

SCAR scale (10): Non-inferiority test showed that the SCAR score of the control group (3.23 ± 1.64) was similar to the observation group (3.07 ± 1.78) (95% confidence interval), and the final scar outcome was similar (*P* > 0.05). (Table 5).

**Table 5:** Comparison of SCAR scores between two groups before and after intervention [scores, ($\bar{x}$±s)].

SCAR evaluation items	Control	Observation	*P*
Observer evaluation			
Scar expansion	0.67 ± 0.23	0.71 ± 0.23	
Erythema	0.61 ± 0.37	0.55 ± 0.21	
Pigment abnormality	0.26 ± 0.11	0.27 ± 0.16	
Surgical trace	0.72 ± 0.25	0.81 ± 0.37	
Hypertrophy/atrophy	0.47 ± 0.21	0.41 ± 0.64	
Overall impression	0.52 ± 0.34	0.49 ± 0.73	
Patient problems			
Pruritus	0.12 ± 0.04	0.09 ± 0.03	
Pain	0.06 ± 0.02	0.05 ± 0.02	
Total score	3.23 ± 1.64	3.07 ± 1.78	0.12

## 4 Discussion

The study findings demonstrate that parents in the observation group using fast-absorbing collagen sutures exhibited significantly lower postoperative anxiety (SAS) and depression (SDS) scores, alongside superior quality of life (GQOLI-74) outcomes compared to the nylon suture control group. This differential effect is mechanistically linked to the suture material's non-removal property. First, traditional nylon sutures require removal on postoperative day 7—a process often accompanied by pediatric distress, wound re-exposure, and procedural discomfort. These factors collectively induce a “secondary trauma” effect in parents witnessing their child's discomfort, thereby amplifying anxiety. In contrast, collagen sutures eliminate invasive suture removal, simplifying postoperative care and reducing psychological burdens associated with complex homecare routines.

Further analysis identifies care protocol simplification as the critical mediator of parental psychological improvement. The observation group required only a single saline cleansing session, whereas the control group necessitated repeated dressing changes and suture removal, increasing both time and economic costs. By minimizing postoperative interventions, fast-absorbing sutures allow parents to focus on holistic caregiving rather than medical procedures, enhancing stress-coping capacity.

The rapid absorption profile and high biocompatibility of collagen sutures may further contribute by accelerating wound healing and reducing infection/scarring risks (11). This biological advantage alleviates parental concerns about complications, reinforcing psychological benefits through a “non-removal → simplified care → risk reduction” cascade. These findings validate the psychosocial relevance of suture material selection and establish a theoretical foundation for optimizing family-centered trauma management strategies. Clinically, this underscores the importance of integrating material science advancements with psychological support frameworks in pediatric care.

Previous studies on suture materials for children's facial trauma have long been confined to a single dimension of technical indicators, mostly focusing on biomedical outcomes such as the wound healing rate, infection rate, and scar score (12). This research paradigm simplifies the management of children's trauma into a purely physiological repair process, severely lacking attention to the impact on family mental health. From the perspective of clinical practice, a large amount of literature has confirmed that there is no significant difference in scar formation or infection risk between nylon sutures and absorbable sutures. However, such conclusions remain only at the physiological level and fail to address the potential impact of the choice of suture materials on the family's care experience. For example, in Zhang's study, by comparing 632 cases of children's facial trauma using nylon sutures and absorbable sutures, it was found that there was no statistical difference in scar scores between the two groups three months after the operation, but it completely ignored the psychological pressure borne by parents during the nursing process (4). The comparable SCAR scores between groups challenge the traditional emphasis on suture material selection based solely on scar-related outcomes. While prior studies have focused on biological metrics like infection rates and scar formation, our findings demonstrate that psychological benefits for caregivers can coexist with equivalent aesthetic results.

In fact, children's facial trauma is not only a physiological trauma for the child but also a stressful event for the entire family (13). As the main caregivers, parents often bear enormous psychological pressure due to concerns such as wound infection, scarring, and pain during suture removal. Although clinical practice has found that dissolvable sutures can simplify the nursing process, the associated mechanism between them and the psychological pressure of parents has never been quantified, leading to a lack of empirical support for the “family-centered” trauma management strategy in clinical practice.

This study systematically reveals for the first time through a randomized controlled trial the direct association between the choice of suture materials and the mental health outcomes of parents, filling the cognitive gap in the field of family psychological intervention. Compared with the traditional research paradigm, the innovation of this study is reflected in the following two aspects: Firstly, it dynamically links the physical properties of suture materials (such as dissolvable without the need for suture removal) with the family's psychological pressure, confirming that the simplification of the nursing process can significantly reduce the anxiety and depression levels of parents. In the experimental design of this study, the experimental group used fast-absorbing collagen sutures, and the control group used traditional nylon sutures. Through a six-week follow-up survey, it was found that the SAS scores and SDS scores of parents in the experimental group decreased, which was significantly better than those in the control group. Secondly, it quantifies the extent and direction of the improvement in family quality of life through multi-dimensional scales (SAS, SDS, GQOLI-74), providing an operable evaluation framework for the “biological-psychological-social” medical model. Specifically, the study comprehensively evaluated the impact of different suture materials on the family quality of life from four dimensions of material life, social function, psychological function, and physical function using the GQOLI-74 scale, and found that the scores of families using fast-absorbing collagen sutures significantly increased in the dimensions of social function and psychological function.

This groundbreaking discovery not only expands the research dimension of suture materials but also redefines the goal of children's trauma management—shifting from a single pursuit of physiological healing to taking into account the overall well-being of the family. It reminds clinical workers that when choosing suture materials, in addition to considering traditional indicators such as the biocompatibility and mechanical properties of the materials, family mental health should also be incorporated into the decision-making considerations. At the same time, the research results also provide new ideas and methods for subsequent related research, promoting the development of the field of children's trauma management towards a more humanized and comprehensive direction.

Despite revealing the positive impact of fast-absorbing collagen sutures on parental psychological status and quality of life, this study has several limitations. First, the research sample was exclusively sourced from a single medical institution with a small sample size, and did not include families from diverse geographic regions or economic backgrounds, which may limit the generalizability of the conclusions. Additionally, the study only enrolled children under 10 years of age without analyzing differential psychological impacts on parents across developmental stages, thereby restricting the external validity of the results.

Second, the observation period was relatively short, assessing psychological changes only within 1 month postoperatively, without tracking long-term effects. For instance, the final scar morphology typically stabilizes 3–6 months post-surgery, and parental concerns about scarring may evolve dynamically over time, but such potential psychological fluctuations were not analyzed. Furthermore, data collection relied on self-reported scales (SAS, SDS, GQOLI-74), which may introduce recall bias or social desirability bias. Future studies should incorporate objective measures (e.g., cortisol levels (14), video analysis of homecare behaviors (15) to enhance credibility. Lastly, the study did not systematically control for confounding factors influencing parental psychology, such as household financial stress, social support levels, or variations in child compliance. These factors may indirectly influence anxiety and quality of life by modulating caregiving difficulty or resource accessibility, yet the current design omitted stratified analyses. Subsequent research should employ multivariate models or subgroup analyses to clarify the independent association between suture material selection and family mental health outcomes. Although care protocols reflected standard practice for each suture type, differential intervention frequency may independently influence parental anxiety. Future studies could control this by using identical non-touch protocols (e.g., Steri-Strips over all wounds) while comparing suture materials.

Notably, this study has several limitations that warrant acknowledgment. First, the research did not account for broader psychosocial factors (e.g., household economic stress, social support networks, or pre-existing parental mental health conditions) that may influence caregiver stress levels and modulate study outcomes. Second, the follow-up period was restricted to 1 month postoperatively, which does not capture long-term scar maturation (typically stabilizing 3–6 months after surgery) or sustained parental psychological adjustments. Future investigations should incorporate longitudinal designs with extended follow-up and multivariate analyses to control for confounding psychosocial variables, thereby enhancing the generalizability of findings across diverse populations and clinical contexts.

## 5 Conclusion

Fast-absorbing collagen sutures, compared to nylon sutures, reduce parental anxiety and depression, improve family quality of life in pediatric facial trauma repair, with no compromise on scar outcomes (assessed by SCAR scale). Their non-removal property simplifies postoperative care, eases procedural distress and repeated intervention burdens, which drives parental psychological improvement. The equivalent SCAR scores indicate suture material selection should consider not only physical outcomes but also caregiver mental health and family well-being. These findings support absorbable sutures as a viable option in pediatric trauma management requiring sutures, potentially enhancing family-centered care via simplified postoperative protocols.

## Data Availability

The original contributions presented in the study are included in the article/Supplementary Material, further inquiries can be directed to the corresponding author.
